# The Molecular Biology of Midgut Neuroendocrine Neoplasms

**DOI:** 10.1210/endrev/bnad034

**Published:** 2023-12-20

**Authors:** Amy P Webster, Chrissie Thirlwell

**Affiliations:** Department of Clinical and Biomedical Science, University of Exeter College of Medicine and Health, Exeter, EX2 5DW, UK; Department of Clinical and Biomedical Science, University of Exeter College of Medicine and Health, Exeter, EX2 5DW, UK; University of Bristol Medical School, University of Bristol, Bristol, BS8 1UD, UK

**Keywords:** neuroendocrine neoplasms, neuroendocrine tumors, genetics, epigenetics, DNA methylation, molecular biology

## Abstract

Midgut neuroendocrine neoplasms (NENs) are one of the most common subtypes of NEN, and their incidence is rising globally. Despite being the most frequently diagnosed malignancy of the small intestine, little is known about their underlying molecular biology. Their unusually low mutational burden compared to other solid tumors and the unexplained occurrence of multifocal tumors makes the molecular biology of midgut NENs a particularly fascinating field of research. This review provides an overview of recent advances in the understanding of the interplay of the genetic, epigenetic, and transcriptomic landscape in the development of midgut NENs, a topic that is critical to understanding their biology and improving treatment options and outcomes for patients.

## Graphical Abstract

**Graphical Abstract:**
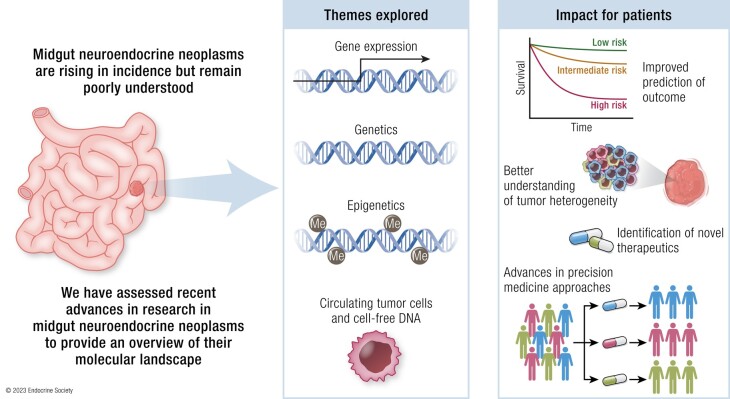

**Essential Points**

- The unusually low mutational burden of midgut neuroendocrine neoplasms (NENs) alongside their slow-growing indolent nature and paradoxical high metastatic potential makes these tumors biologically intriguing.
- The most commonly identified genetic aberrations in these tumors are chromosome 18 loss of heterozygosity (LOH) and mutations in *CDKN1B*, though recent genetic analyses have identified less common mutations that provide potential therapeutic candidates.
- Recent studies have demonstrated that multifocal midgut NENs evolve independently, indicating some kind of cancer priming factor may be playing a role in tumorigenesis.
- The majority of epigenetic variants identified in midgut NENs appear to be related to chromosome 18 LOH status, and cellular composition analyses based on DNA methylation data have identified enrichment of CD14 infiltration in non-18 LOH tumors, which correlates with lower progression-free survival.
- Gene expression analyses have identified differences in expression of genes that correlate with treatment response or survival outcomes, which has been exploited for the development of liquid biopsies, which can inform treatment strategy, such as the NETest.

## Background

Neuroendocrine neoplasms (NENs) are tumors that develop from the cells of the nervous and endocrine systems. They are relatively rare, though their incidence is rising globally (1-3), with a 6.4-fold increase in cases reported in the United States in recent decades (2). Midgut NENs, also referred to as small intestinal or ileal NENs, are thought to account for around 18% of NEN diagnoses (1), making them one of the most common subtype of NENs, and are the most commonly diagnosed malignancy of the small intestine (4). Midgut NENs are typically slow-growing and indolent tumors, though they have a high metastatic potential with around 76% of patients presenting with metastases at diagnosis or during follow-up (1). While the risk of metastasis correlates with the size of the tumor, even very small tumors of less than a centimeter can metastasize (5). Small intestinal metastases account for around 27% of all cases of NEN metastases, accounting for more metastases than lung or pancreatic primaries; however, they are also associated with better survival (6). This juxtaposition between the indolent nature of midgut NENs and their propensity to cause metastatic disease makes them intriguing. Although the incidence of NENs is increasing, it has been noted that the rate of diagnosis of metastatic disease has remained stable over the past 50 years (2, 7). It has been suggested that this trend could be due to improved identification of asymptomatic, early-stage disease through increased use of endoscopic and imaging procedures (2). In addition, survival of patients with metastatic NETs has improved, due to earlier diagnosis and the use of systemic therapies (2).

While many cases of midgut NENs are sporadic, an increase in incidence has been noted among relatives of patients diagnosed with the disease (8). Some large epidemiological studies have demonstrated that in familial cohorts of midgut NENs the relative risk was between 2.8 and 4.3 (9-11). Several genes involved in DNA base excision repair have been associated with familial midgut NENs; however, to date each mutation has been uniquely identified in single families (12). In a whole-exome sequencing study of 33 families, each with at least 2 cases of midgut NENs, a germline deletion in the *IPMK* gene was identified, which caused reduced kinase activity and nuclear localization, consequently impacting on the activation of p53 (13). Familial midgut NENs are usually asymptomatic (8) and subsequently are often not diagnosed early, indicating the importance of improved prediction and potentially screening for family members.

## Mutational Landscape of Midgut NENs

Midgut NENs are mutationally quiet compared to other solid tumors (14), and the majority do not harbor a candidate driver gene mutation (15). As sequencing technology has advanced, the identification of molecular markers has become more refined, and recent efforts using whole-genome sequencing approaches have improved our understanding of the spectrum of mutations present in these tumors.

Several sequencing studies have identified that midgut NENs have a relatively stable genome, with an average of just 0.1 single nucleotide variant per 106 nucleotides (14, 16).

*CDKN1B*, a gene that encodes the p27 cell cycle regulator, is known to be the most frequently mutated gene in midgut NENs (16-18) and is believed to be a distinct driver gene associated with the disease. However, this gene has been found to be mutated in only around 8% of cases (16, 19), indicating that other mutations or alterations in epigenetic gene regulation are key in driving development of midgut NENs. Interestingly, this gene was found to be mutated more frequently in a study focussed on metastatic disease, where it affected 23% of tumors (17), indicating this mutation is possibly associated with more aggressive disease phenotype in midgut NENs. Another study that analyzed multiple midgut NEN and metastatic tumors from the same individuals found that the *CDKN1B* mutation often occurred quite late in tumor development. In 2 patients, the *CDKN1B* mutations were identified in metastatic primary tumors but were absent from the corresponding metastases, indicating the mutation happened after metastasis had occurred. In these instances, it appears that though *CDKN1B* is present in the tumor, it is not acting as a driver mutation and is not necessary for metastasis (20).

## Using the Genomic Landscape of Midgut NENs for Novel Therapeutic Identification

As well as acting as biomarkers of disease subtype and prognosis, understanding the mutational landscape of these tumors can also give insight into potential novel therapeutics. A recent study that performed whole-genome sequencing of 85 neuroendocrine neoplasms (including 39 midgut NENs) found that 49% of samples contained at least 1 mutation in a known drug target gene that has a treatment agent either already available or currently in development (17). Just 26% (n = 10) of midgut-derived advanced neuroendocrine tumors (aNETs) contained actionable mutations, comparatively lower than the 55% of the pancreas-derived aNETs containing actionable mutations. There also appeared to be heterogeneity across midgut-derived aNETs with most mutations identified in only 1 tumor. The 3 exceptions to this were *KRAS*, *RB1,* and *MTAP,* which each appeared in 2 midgut-derived aNETs, indicating they may be more common mutations (albeit in a limited sample size) and potentially better candidates for therapeutic development. It is important to note that as this study focused on advanced disease, samples were likely to be enriched for actionable mutations. In addition, the majority of these tumors were sampled from metastases, rather than the primary tumors, again biasing the findings toward more severe metastatic disease and making findings potentially only relevant to treatment of metastatic tumors. Despite these biases, identification of such genetic loci could allow development of and repurposing of therapies for the treatment of midgut NENs, which currently have limited effective treatment options available. However, this approach is currently not utilized in routine clinical care at this time and would only be a viable option for routine care when the cost of whole-genome sequencing reduces dramatically or if further research identifies a selection of several genes with utility for treatment selection that could be used in a candidate gene sequencing panel.

While many studies have determined mutational burden and distribution as an average across the samples included in the study, midgut NENs have been found to be highly genetically heterogeneous (17, 21). This genetic heterogeneity can hinder the interpretation and understanding of the tumor biology and indicates the importance of considering multiomic effects on gene deactivation simultaneously to gain a more complete understanding of midgut NEN development.

## Nonclonal Origin of Multifocal NENs

Typically during cancer development, a primary tumor forms, which may release cells that circulate and attach to other tissues in the process we know as metastasis. These metastatic tumors usually retain a similar mutational signature to their parent tumor. An unusual aspect of small intestinal NENs is that multiple separate synchronous tumors seem to develop throughout the small intestine and are termed “multifocal.” The majority of research into midgut NENs to date has focused on single primary tumors; however, up to 50% of cases present with multiple primary tumors (22-24).

**Figure 1.**
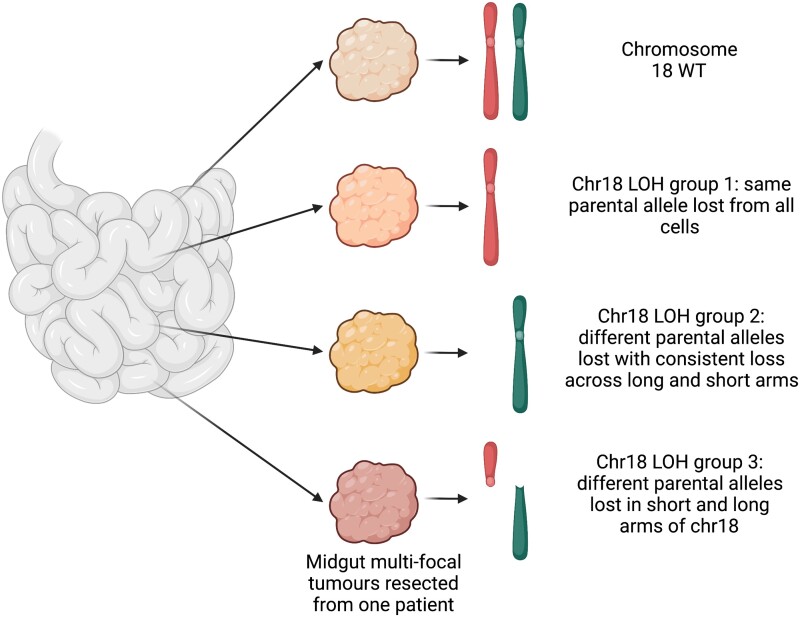
Patterns of chromosome 18 loss across multifocal midgut neuroendocrine neoplasms (adapted from Zhang et al 2020; created with BioRender).

These multifocal tumors were originally assumed to be clonal; however, several recent studies have set out to investigate the intrapatient genetic variability of multifocal tumors. These studies have consistently demonstrated that these tumors originate independently. This was shown initially through PCR methods testing for chromosome 18 loss of heterozygosity (LOH) (25) and more recently using sequencing methods exploring copy number variation (26) and whole-genome single nucleotide variation (20). Zhang et al identified 3 patterns of chromosome 18 LOH, finding that tumors could lose the same or different parental alleles or could also lose different alleles in the long and short arms of the chromosome. They identified multiple patterns occurring in the same patient, including 1 patient who had tumors representing each of the 3 patterns in addition to tumors with wild-type presentation of chromosome 18 (as illustrated in Fig. 1). A more recent study, which performed whole-genome sequencing on 61 tumors from 11 patients with multifocal lesions, found a lack of shared somatic single nucleotide variants between tumors (20). They also identified that metastatic tumors within individuals originated from multiple primary tumors, indicating that multiple tumors had independently gained metastatic potential during development. These studies also agree with previous findings that chromosome 18 LOH is the most common genetic alteration in midgut NENs (26, 27) and that *CDKN1B* mutations are the most frequent somatic aberration (16, 20) in midgut NENs, regardless of whether they are multifocal or not.

Multiple endocrine neoplasia type 1 (MEN1) is a disorder caused by mutations in the *MEN1* gene, which encodes a tumor suppressor protein called menin. Mutations in the MEN1 gene results in abnormal menin protein production, which leads to MEN1 syndrome, demonstrated by development of multiple NEN tumors. While the studies described earlier focused on patients who have no known genetic syndrome, germline mutations in *MEN1* can increase susceptibility to multifocal primary NENs, and such mutations have been identified in midgut NENs (28), though this is rare.

The genetic variation between tumors within a single patient indicates independent somatic evolution, which presents a biological quandary as to why these multiple tumors develop in the absence of an inherited genetic syndrome. Previous theories around the development of these tumors have included exposure of the tissue to a local “cancer-priming” factor (20); however, further research is needed to substantiate this. While recent advances have greatly improved our understanding of multifocal NEN phylogeny and evolution, further research into the causes of multifocal NEN development is needed to give insight for potential prevention and improved treatment strategies. Such insight may be gained from understanding the regulatory landscape of the multifocal midgut NEN genome.

## Epigenetic Landscape of Midgut NENs

The low mutational burden of midgut NENs, along with the lack of consistent driver mutations, indicate that other factors may be playing a key role in driving tumor development. One factor that has been explored as a potential driver of tumorigenesis is epigenetics, in particular DNA methylation.

In a study by Karpathakis et al, DNA methylation was found to distinguish midgut NENs into 3 molecular subgroups of prognostic significance (29): group 1 characterized by chromosome 18 LOH, group 2 containing no copy number variation, and group 3 harboring multiple copy number variations. Though these subgroups were identified using DNA methylation arrays, they are defined by copy number alterations rather than true epigenetic differences. Further work in this area has identified differentially methylated genes that are evident between the 3 molecular subgroups. When comparing tumors with chromosome 18 LOH with the other 2 groups, 901 genes were found to be differentially methylated with a particular enrichment for genes from the G-protein coupled receptor pathway (30). These differentially methylated genes could give insight into potential therapeutic pathways, demonstrated by the differential methylation of the somatostatin (*SST*) gene. When the promoter of *SST* is hypermethylated in midgut NENs, the somatostatin protein has reduced expression and consequently reduced the ability to inhibit tumor growth. The receptor of this protein, somatostatin receptor (SSTR) 2 is the target of an Food and Drug Administration-approved theranostic. This study also identified multiple differentially methylated genes, which correlated with survival outcomes within the chromosome 18 LOH subgroup, including *TRHR*, another gene within the G-protein coupled receptor pathway that is the target of several Food and Drug Administration-approved therapies (30).

A more recent study by Waterfield et al further investigated these subgroups of midgut NENs in a multiomics approach. When subtypes of midgut NENs were compared, a general trend of lower methylation in chromosome 18 LOH tumors was observed, and several genes associated with chromosome 18 LOH status were found to have disrupted methylation patterns (31). Combined multiomics analysis using DNA methylation and gene expression data identified 12 genes with higher expression in chromosome 18 LOH tumors, which also had an enrichment of differentially methylated CpG sites. This indicates that the differential methylation observed is influencing gene expression in these tumors. When regional methylation was assessed, 3 genes were highlighted as significantly different between chromosome 18 LOH and non-18 LOH groups (*SCRT1*, *AMPD3,* and *GRAMD2*). Interestingly the *AMPD3* pathway was also independently identified using the SMITE software for combined analysis of expression and methylation data. This gene is involved with purine metabolism, which has previously been associated with progression of cancer (32).

The same study also utilized the cell-specific patterns of DNA methylation to impute cell type composition within primary midgut NEN tumors and identified specific patterns of cell composition with prognostic significance. Specifically, in midgut NENs that lack chromosome 18 LOH, this study found that the tumors contained higher levels of CD14 infiltration, which corresponded with lower progression-free survival.

A comparison of DNA methylation across multiple types of NEN found that small intestinal NENs had the highest proportion of differentially methylated positions, and these NENs clustered separately from all other NENs tested (33), indicating that the epigenetic landscape of midgut NENs is distinct from NENs originating in other organs. This is an important consideration for future study design, as due to the rare nature of NENs it is not uncommon for multiple categories of NEN to be analyzed together to increase statistical power, though these findings demonstrate that this is an inappropriate approach when assessing the molecular biology of midgut NENs.

A further application of epigenetics in NEN diagnosis and treatment has been the use of DNA methylation profiles to identify NENs of unknown primary origin. A significant proportion of NENs present with metastases at diagnosis, and the primary tumor is unknown in approximately 1 in 9 cases of NENs (34). Due to the underlying differences in molecular biology of NEN tumors originating from different origins, this has implications for treatment decisions and prognosis. Recently, a tool has been developed using machine learning to exploit the tissue-specific methylation differences in NEN tumors to allow identification of primary origin in such tumors (35). This has been developed for identification of tumors originating from the small intestine, pancreas, and lung. While validation and expansion to include other tissues of origin is needed, this represents a promising step forward for the identification of unknown primaries in NEN diagnosis, which would improve decisions about most appropriate treatments and also identification of small, previously undiagnosed primary tumors.

## Transcriptomic Differences in Midgut NENs

Studies of the gene expression in midgut NENs have identified that low expression of *RASSF1A* and *P16* in the tumors correlated with poor survival (36). Interestingly, in a separate study of DNA methylation, the *RASSF1* gene was also found to be methylated more frequently in metastatic tumors (37), indicating the expression-linked differences in patient outcome are mediated by epigenetic modifications.

## Liquid Biopsies in Midgut NENs

Circulating RNA has also been used to develop a test for neuroendocrine tumor gene expression, called the “NETest” (38). The test uses a panel of 51 NEN-specific marker genes to predict tumor activity and a “risk index” for each patient, as well as correlating with clinically relevant outcomes such as response to somatostatin analogs and peptide receptor radiotargeted therapy. The NETest has the potential to improve the diagnosis, monitoring, and prediction of NENs and to inform management of the disease (38). This has been demonstrated in a trial of the NETest in a cohort of 100 NEN patients, which found it had a 96% diagnostic accuracy (39) and high correlation of the NETest score with disease activity; however, further clinical validation in prospective clinical trials is ongoing.

A more recent assessment of the NETest also found that in a larger cohort of NENs (including 512 midgut NENs), it outperformed the commonly used biomarker chromogranin A (40). In midgut NENs the NETest was found to have an accuracy of 94%, and was able to stratify disease status, differentiating between progressive and stable disease, and was also found to predict the recurrence of tumors following surgery. This study found the NETest was strongly correlated with imaging, which has been proposed as a potential cost-effective method to reduce the radiation exposure associated with diagnostic imaging (39).

However, an independent study investigating circulating tumor DNA (ctDNA) from a range of NENs found that only 24% of midgut NENs had detectable levels of ctDNA, compared to 62% of pancreatic NENs (41). This low level of ctDNA was proposed to be due to differences in tumor biology or location, which may indicate that tests such as the NETest that rely on detection of circulating RNA may be less effective in midgut NENs than other neuroendocrine malignancies. Interestingly, the ctDNA study by Boon et al described earlier also found that presence of detectable levels of ctDNA correlated with treatment status (41), indicating the importance of consideration of treatment history when using measurements from liquid biopsies for therapeutic guidance.

SSTRs are known to be overexpressed in NENs, with *SSTR2* and *SSTR5* being highly expressed in midgut NENs (42), particularly in low-grade well-differentiated NENs. Although most patients do not have uniform high expression of SSTRs throughout their tumors, the expression of these SSTRs has been measured in circulating tumor cells from midgut NEN patients (43), and a recent clinical trial called the “CALM-NET” study has tested if the effectiveness of SSTR targeting therapies can be predicted from circulating tumor cells (44). The authors found that patients treated with a somatostatin anolog (Lanreotide autogel) were more likely to have a positive response to the therapy if they had no detectable circulating tumor cells at baseline, though these results were not statistically significant due to the limited sample size (44). This indicates that measurement of circulating tumor cells could provide a novel and minimally invasive method of optimizing therapeutic selection in the treatment of midgut NENs.

## Prognostic Associations With Chromosomal Loss and Instability

Genetic and epigenetic studies have consistently identified LOH of chromosome 18 as the most common large-scale genetic change in midgut NENs, occurring in approximately 50% of cases (18). This LOH at chromosome 18 has been cited as an indicator of good prognosis in midgut NENs (45). The 3 subgroups identified in the previously mentioned analysis of DNA methylation in midgut NENs by Karpathakis et al (29) were found to correlate with different progression-free survival outcomes following resection of primary tumors, with the chromosome 18 LOH group being associated with more favorable progression-free survival outcomes. This is supported by association with increased progression-free survival in gastrointestinal NENs compared with patients who have both copies of chromosome 18 (18), though this trend was not statistically significant when midgut NENs were analyzed separately.

Chromosomal instability has been observed across all types of NENs, with midgut NENs displaying the highest rate of instability (18). The level of chromosomal instability has been found to be prognostic in midgut NENs. In a large-scale analysis of the RADIANT clinical trials, lower levels of chromosomal instability were found to be associated with longer survival in midgut NENs (18).

## Future Directions and Emerging Concepts in Midgut NEN Molecular Biology

A recent study explored the use of next-generation sequencing in peripheral blood samples, liquid biopsies, and formalin-fixed paraffin-embedded samples of NEN tumors to test for mutations that could inform treatment pathways (46). While peripheral blood (which would indicate only germline mutations) indicated 16 variants predisposing to NEN development in 50% of patients tested, the novel aspect of the study was its use of liquid biopsies. Somatic mutations in the DNA derived from tumors (in liquid biopsies and formalin-fixed paraffin-embedded samples) were found to be informative for treatment selection (ie, indicating treatment sensitivities in the tumors) in 5 of the 9 tumors tested. Notably, the majority of these mapped to the *RET* oncogene. While this is a very small study requiring validation, it demonstrates the potential for using molecular biology to select therapies in a precision medicine approach to NEN treatment. In the context of liquid biopsies, this provides a powerful way of testing for such variants that would greatly improve treatment outcomes for patients in a noninvasive and cost-effective method.

**Figure 2.**
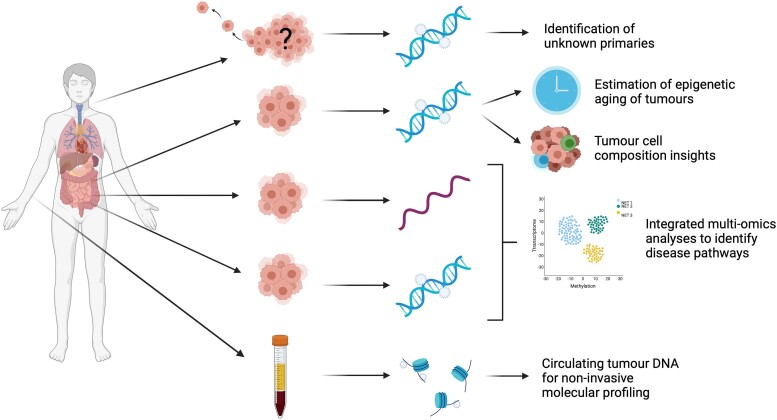
Emerging concepts in midgut neuroendocrine neoplasms molecular analyses. Figure created with BioRender.

Another challenge has been the determination of how long a tumor has been present in the tissue and, in the case of multifocal tumors, which tumors had developed first. Due to the slow-growing and indolent nature of the majority of midgut NENs, the size of the tumor is not particularly informative in this regard. However, advances in epigenetic analysis techniques of DNA methylation data have allowed the development of epigenetic “clocks” that exploit the progressive changes in DNA methylation that reflect natural aging (47, 48). These clocks have demonstrated accelerated ageing phenotypes in cancer tissue (47, 49, 50), the degree of which may be used to determine the approximate “age” of tumors (Fig. 2). While this technology has yet to be applied to midgut NENs, it has the potential to shed light on the order in which midgut NENs have formed, which may give insight to the underlying cause of these tumors, which remains something of an enigma. Several other DNA methylation-based predictors of exposures and cellular characteristics including metabolic traits have been developed in recent years (51), and, while these tools have been developed in blood, they may also have the potential to provide insight into cellular characteristics and exposures in tumor tissue. Such insight could help us understand the underlying cause of NEN development and potentially further our understanding of tumor progression in midgut NENs. Emerging predictors of levels of inflammatory biomarkers from DNA methylation (52) could also have a valuable application in prediction of NEN prognosis due to the known impact of inflammation on prognosis (53).

Large studies of other cancer types, such as the TRACERx study, which focuses primarily on lung cancer, have demonstrated the possibilities of in-depth genomic and epigenomic profiling for understanding tumor heterogeneity. The cells contained within a tumor and the degree of tumor cell heterogeneity can both give substantial insight into tumor biology and prognosis for the patient as well as allow improved treatment decisions. Tools allowing prediction of cell composition from DNA methylation data make this possible using existing datasets from NEN bulk tissue along with reference datasets from purified cell types present in tumors (54, 55). While there are several limitations of such methods currently, primarily the lack of appropriate reference datasets specifically developed for investigation of NENs, they have the potential to detect tumor-composition patterns in midgut NENs in the future.

Another research technique that could provide insight into heterogeneity in NENs is single-cell DNA and RNA sequencing. Single-cell RNA sequencing has been used effectively in pancreatic NENs (56) and improved understanding of transcriptional states and spatiotemporal dynamics in tumors, which could allow identification of novel markers of prognosis. While this technique provides insight into genomic variation across individual cells within a tumor and a more refined understanding of heterogeneity within a tumor, the technique also presents several challenges including missing data and low data quality (57), which could hinder its application in rare cancers such as NENs. Single-cell sequencing was used in a small group of midgut NENs (n = 4) (58) to assess whether copy number variation analysis could be assessed from circulating NEN cells. The study identified copy number heterogeneity between and within patients that could not be assessed using bulk tissue, demonstrating the potential clinical utility of such techniques. While such analyses are in their infancy, in the future they could prove to be a valuable tool to understand some of the unknowns of midgut NEN biology and could be used to provide a “ground truth” validation of bioinformatic cell deconvolution methods relying on reference datasets such as those described earlier.

## Conclusion

Over the past 10 years through the use of sequencing and array-based molecular analyses we have seen an improvement in our understanding of the molecular biology of midgut NENs, leading to the identification of molecularly distinct subgroups of NEN, the identification of the primary site in NENs of unknown primaries site, and the application of circulating molecular analyses such as the NETest. However, this knowledge has not significantly altered clinical management, guidelines, or treatment algorithms. There is still much to understand and learn about this intriguing NEN subtype, in particular the aetiology of multifocal tumors and inheritance of familial midgut NENs. Ongoing work in these areas will hopefully lead to earlier detection and screening of higher-risk populations in the future.
